# Could the Heat Shock Proteins 70 Family Members Exacerbate the Immune Response in Multiple Sclerosis? An in Silico Study

**DOI:** 10.3390/genes11060615

**Published:** 2020-06-03

**Authors:** Luigi Chiricosta, Agnese Gugliandolo, Placido Bramanti, Emanuela Mazzon

**Affiliations:** IRCCS Centro Neurolesi Bonino Pulejo, 98124 Messina, Italy; luigi.chiricosta@irccsme.it (L.C.); agnese.gugliandolo@irccsme.it (A.G.); placido.bramanti@irccsme.it (P.B.)

**Keywords:** multiple sclerosis, brain, heat shock proteins, immune system, in silico analysis, transcriptome

## Abstract

Multiple sclerosis (MS) is a chronic autoimmune demyelinating disease of the central nervous system. It represents one of the main causes of neurological disability in young people. In MS, the autoimmune response is directed against myelin antigens but other possible bio-molecular markers are investigated. The aim of this work was, through an in silico study, the evaluation of the transcriptional modifications between healthy subjects and MS patients in six brain areas (corpus callosum, hippocampus, internal capsule, optic chiasm, frontal and parietal cortex) in order to identify genes representative of the disease. Our results show the upregulation of the Heat Shock Proteins (HSPs) *HSPA1A*, *HSPA1B*, *HSPA7*, *HSPA6*, *HSPH1* and *HSPA4L* of the HSP70 family, among which *HSPA1A* and *HSPA1B* are upregulated in all the brain areas. HSP70s are molecular chaperones indispensable for protein folding, recently associated with immune system maintenance. The little overexpression of the HSPs protects the cells from stress but extreme upregulation can contribute to the MS pathogenesis. We also investigated the genes involved in the immune system that result in overall upregulation in the corpus callosum, hippocampus, internal capsule, optic chiasm and are absent in the cortex. Interestingly, the genes of the immune system and the HSP70s have comparable levels of expression.

## 1. Introduction

Multiple sclerosis (MS) is a chronic autoimmune disorder that affects the central nervous system (CNS). MS onset normally occurs in young adults, in the range 20–40 years of age, with women more frequently affected compared to men, with a ratio of 3:1 [1]. The disease course can be different between individual patients. Traditionally, MS is classified in different clinical phenotypes: relapsing–remitting MS (RRMS), secondary progressive MS (SPMS), primary progressive MS (PPMS). In RRMS, reversible episodes of neurological deficits, indicated as relapses, characterize the initial phases of the pathology. Later, the development of permanent neurological deficits and the progression of clinical disability become prominent, leading to SPMS. PPMS is present only in a minority of patients that show a progressive disease course since MS onset [1]. The cause of MS is not completely clear, but a multifactorial cause is accepted, where genetic as well as environmental factors may influence the individual disease risk in a complex manner that is not fully clarified. An early treatment of MS is necessary to limit the permanent damage to the CNS. Disease-modifying therapies are considered as standard treatments for MS, and their safety profile is considered to be acceptable [2].

Neuroinflammation, demyelination and neuronal loss occur from the first stages of the disease. 

Immune system plays a main role in the disease. Several studies suggest that the early phases of MS are mainly driven by peripheral immune responses targeting the CNS. Conversely, in the progressive phase, the immune reactions are mainly driven within the CNS.

MS is based on autoimmunity and the myelin antigens are the targets in the disease. Both CD4+ and CD8+ lymphocytes participate, while autoantibodies can have a secondary role or can enhance the process. Autoreactive T lymphocytes against myelin components are also present in normal subjects where they do not induce disorders. Instead, MS is triggered by the induction of pathogenic Th17- and Th1-type and CD8+ autoreactive T lymphocytes directed against myelin components. In addition, both microglia and macrophages accumulate in the areas of active demyelination and neurodegeneration in MS [3]. Nowadays, transcriptional studies can help to understand impaired pathways in the disease. The Heat Shock Proteins (HSPs) are families of proteins produced inside the cell as result of stressful conditions. They are mainly distinguished by their molecular weight and the HSP70s (70kDa) play a primary role in neurodegenerative diseases. Indeed, HSP70s’ overexpression was associated with a protective role in stroke, epilepsy, Alzheimer’s and Parkinson disease [4]. Conversely, there are discordant opinions about autoimmune diseases like MS. Indeed, a small upregulation of the HSPs can be associated with a protective role but a strong upregulation of HSPs can act as a warning signal that triggers the immune system [5]. Thus, the pathways affected by HSPs alteration can have implications in the pathogenesis of MS and are the subject of studies [6,7]. 

The aim of this work was to identify altered patterns of gene expression in different areas of the brain of MS patients compared to healthy subjects (corpus callosum, hippocampus, optic chiasm, internal capsule, frontal cortex and parietal cortex). For this reason, through an in silico analysis, we compared the transcriptional profile of MS and healthy subjects in order to evaluate the genes that simultaneously intervene in the different areas. Moreover, we evaluated the most affected pathways and their biological significance. 

## 2. Materials and Methods

### 2.1. Data Collection

The in silico analysis was performed using the Gene Expression Omnibus (GEO) repository [8] freely available by the National Center for Biotechnology Information (NCBI) [9]. We retrieved the data deposited by Voskuhl et al. from the accession codes GSE123496 and GSE100297 [10]. We selected five runs of healthy subjects and five runs of MS patients from the Sequence Read Archive repository [11] for six different brain areas (corpus callosum, hippocampus, optic chiasm, internal capsule, frontal cortex and parietal cortex). The tissues were obtained from five female MS patients (average age = 57.6 years) and five female healthy subjects (average age = 56.2 years). Tissues did not show lesions by neuropathology with the aim of evaluating gene expression in normal appearing white matter and normal-appearing gray matter. MS types included one RRMS with transition to SPMS, three progressive, one unknown. Disease-modifying therapies were not used in 4, while unknown in 1. Optic neuritis was confirmed in 1, and it is unknown in 4.

### 2.2. In Silico Analysis

The quality of the raw data was confirmed by fastQC. We retrieved the data in “Fastq” format, and we trimmed the sequences using Trimmomatic (Usadel Lab, Aachen, Germany) [12] (version 0.38) (LEADING: 30 TRAILING: 28 SLIDINGWINDOW: 4:28 MINLEN: 75). The reference genome of the “Homo Sapiens” (GRCh37), freely available from the University of California Santa Cruz (UCSC) web site [13], was used to support the reads’ alignment and sorting performed by Spliced Transcripts Alignment to a Reference (STAR) RNA-seq aligner [14]. For each sample, the count of the reads was performed using htseq-count [15] under python (version 2.7.15, Python Software Foundation, Wilmington, DE, USA). Finally, the changes in the expression profile between healthy and MS subjects were evaluated with DESeq2 [16] in R. We did not use any cutoff for the fold change but we performed the post-hoc correction of the *p*-value using Benjamini–Hochberg procedure with a threshold < 0.05 to filter out the false positives and define the differentially expressed genes. The full code used for the analysis can be found in Table S1. Moreover, in order to study the genes involved in the immune system, we took advantage of the database KEGG, and in particular we focused on the pathways “Antigen processing and presentation”, “B cell receptor signalling pathway”, “T cell receptor signalling pathway” and “Th17 cell differentiation” [17].

## 3. Results

### Transcriptomic Analysis of MS Patients and Healthy Subjects

**Figure 1:**
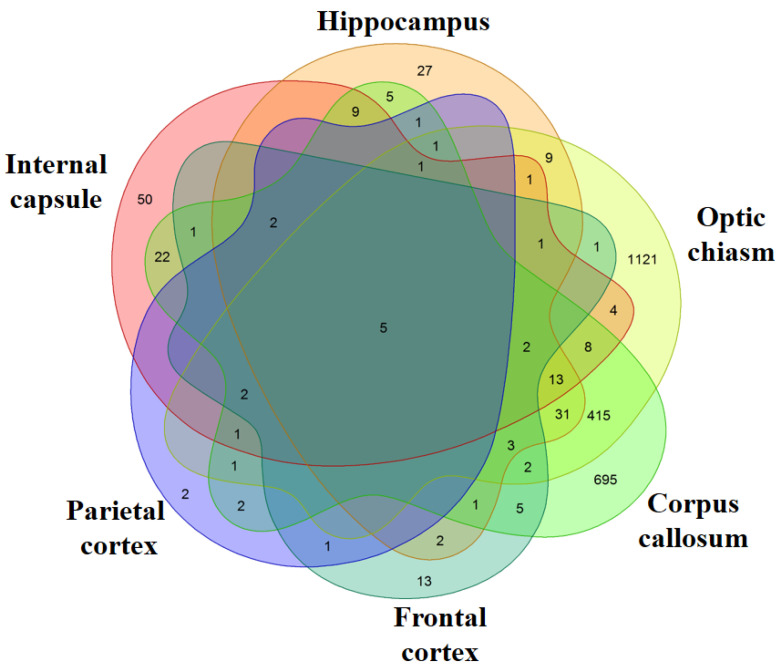
Venn Diagram. For each area, the genes differentially expressed in healthy against multiple sclerosis (MS) subjects were inspected. The comparison of all the areas shows how many genes are differentially expressed between the different areas. Each intersection of the different areas shows the number of genes in common exclusively between those groups and not with the others. The most of the differentially expressed genes are in Optic chiasm and Corpus callosum while very few genes are expressed in the Frontal and Parietal cortex.

We evaluated the genes differentially expressed in the six different brain areas. In particular, we compared each area of the MS patients against the correspondent area of the healthy subjects. The analysis of the corpus callosum revealed the presence of 1228 differentially expressed genes (651 upregulated and 577 downregulated) in MS patients compared to healthy subjects (Table S2). Instead, in the hippocampus, 114 genes are differentially expressed (93 upregulated and 21 downregulated) (Table S3). The differentially expressed genes in optic chiasm are 1622 (548 upregulated and 1074 downregulated) (Table S4). In the internal capsule, 122 genes are differentially expressed (96 upregulated and 26 downregulated) (Table S5). The cortexes have the lowest number of genes differentially expressed and, specifically, 41 genes for the frontal cortex (32 upregulated and 9 downregulated) (Table S6) and 19 for the parietal cortex (all upregulated) (Table S7). Except for the optic chiasm, the most of the differentially expressed genes in the inspected areas are upregulated. The Venn Diagram in Figure 1 shows the genes differentially expressed between the healthy subjects and the MS patients and the number of differentially expressed genes that are in common between the different areas. In particular, each intersection of the different areas shows the number of genes in common exclusively between those groups and not with the others. The diagram highlights that the optic chiasm and the corpus callosum are the most involved areas. Nevertheless, the five transcripts *HSPA1A*, *HSPA1B*, *DNAJB1*, *CHORDC1*, *CTB-3601.7* are differentially expressed in all the brain areas and, interestingly, they are always upregulated. Except for *CTB-3601.7,* which is an uncharacterized gene, *HSPA1A*, *HSPA1B*, *DNAJB1* belong to the HSPs, while *CHORDC1* ontology reveals its association with HSPs.

**Figure 2:**
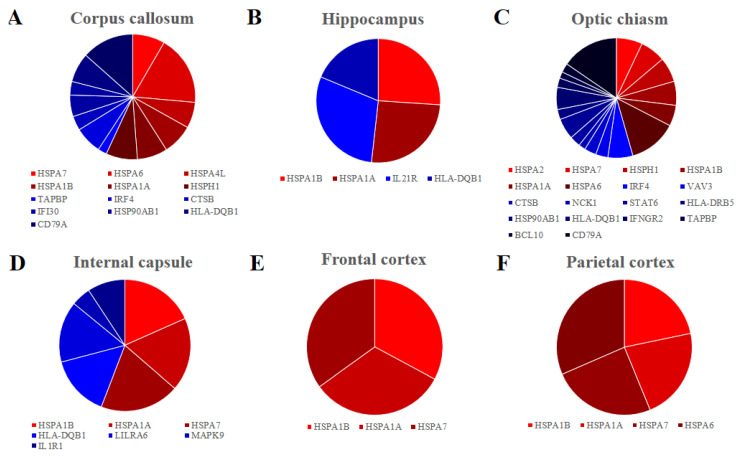
Pie chart of the fold change distribution between the genes that belong to the HSP70s (red palette) and the genes that are involved to the Immune system (blue palette) in the different brain areas. Corpus callosum (**A**), Hippocampus (**B**), Optic chiasm (**C**) and Internal capsule (**D**) have comparable level of fold change between the genes that are involved in immune system and HSP70s. Frontal (**E**) and Parietal (**F**) cortex express only genes encoding for the HSP70s.

Furthermore, we inspected in all the areas the genes encoding for HSP70s, the most studied group of the HSPs and they are listed in Table 1. Overall, seven genes encoding for HSP70s are differentially expressed *HSPA1A*, *HSPA1B*, *HSPA7*, *HSPA6*, *HSPH1*, *HSPA4L* and *HSPA2*. They are upregulated in all the tissues except for *HSPA2* in the optic chiasm. Interestingly, *HSPA1A* and *HSPA1B* are expressed in all the samples. We also inspected our data with KEGG database to find genes that take part in the immune system. Specifically, we listed in Table 2 the genes of our dataset that are included in the pathways “Antigen processing and presentation”, “B cell receptor signalling pathway”, “T cell receptor signalling pathway” or “Th17 cell differentiation”. We found 24 upregulated genes, among which seven are in corpus callosum, two in hippocampus, 12 in optic chiasm and three in internal capsule. Only one gene (*MAPK9*) is downregulated and it is expressed in the internal capsule. No gene was found to be deregulated in the frontal or parietal cortex. Finally, in Figure 2, we represented the distribution of the genes involved in HSP70s against the genes involved in the immune system paying attention to the expression level of each gene for each of the brain areas.

## 4. Discussion

MS disease is characterized by axonal demyelination both in the brain and spinal cord that can be associated with neuronal damage. However, different mechanisms are involved in the axonal degeneration, including neuroinflammation, breakdown of the blood-brain barrier and reactive gliosis. The immune system seems to play a main role in MS pathogenesis. Nevertheless, other environmental and genetic factors could be implicated in the origin and in the progression of the disease. 

In this study, we put under investigation the genes that are differentially expressed in six brain areas corpus callosum, hippocampus, optic chiasm, internal capsule, frontal cortex and parietal cortex. Specifically, we compared the brain tissues of the healthy subjects against MS patients for each area. Our results show that all the brain areas share the HSP70s *HSPA1A* and *HSPA1B* (Figure 1). To date, scientific literature reports several genetic analyses based on HSP70s polymorphisms that associate the HSPs with MS pathogenesis. This evidence suggests that the genes that encode for HSP70s have also a regulatory role in MS.

In particular, genetic studies of polymorphisms in genes that encode for HSPs highlighted an increase in risk and susceptibility to MS [18]. For instance, the homozygous polymorphism rs2227956 in the gene *HSPA1L* seems to increase the risk of developing MS by seven times. This polymorphism alters the functional activity of the protein and leads to the accumulation of misfolded proteins into glia and neurons, promoting autoimmune and inflammatory responses [19]. The +1267 A/G polymorphism of the *HSPA1B* gene seems to increase the susceptibility to MS if the gene is inherited recessively. Consequently, it reduces the expression of the protein Hsp70-2 that could contribute to counteracting the oxidative stress [20]. Nevertheless, most of the associations about HSPs polymorphisms and MS are weak, as shown in other studies related to Iranian [21] and Japanese [22] populations. Beyond the genetic studies, the HSPs seems also to play a regulatory role in MS. It was shown that patients with MS have significantly higher Hsp70 serum levels compared to healthy individuals [23]. Different studies also found significant differences in the expression of HSPs in the cells and tissues of MS patients compared to healthy individuals [24,25]. For this reason, instead of focusing on polymorphisms, we investigated the expression of the genes that encode for the HSP70s for all six brain areas that we studied (Table 1). We identified seven genes differently expressed: *HSPA1A*, *HSPA1B*, *HSPA7*, *HSPA6*, *HSPH1*, *HSPA4L* and *HSPA2*. 

*HSPA1A* and *HSPA1B* are paralog genes that encode for two almost identical isoforms of Hsp70 protein, one of the most studied stress-inducible HSP70s. Through its chaperone activity, Hsp70 exerts a cytoprotective role and hinders multiple steps of the apoptosis [26]. Nevertheless, Hsp70 seems to play a double role, depending on its localization. Indeed, Mansilla M.J. et al. have already studied Hsp70 both in vitro and in vivo and observed a reduced development of experimental autoimmune encephalomyelitis in the animals deficient in Hsp70 [27]. According to another work of Mansilla M.J. et al., the extreme upregulation of Hsp70 cannot prevent the cell death and, consequently, it is released in the milieu where it triggers a vast immune response by cytokines that can damage myelin [28]. Our results show that all the brain areas that we studied—corpus callosum, hippocampus, optic chiasm, internal capsule, frontal cortex and parietal cortex—overexpress both *HSPA1A* and *HSPA1B*. Interestingly, they are the only genes in our analysis that are overexpressed in all the samples. The genes *HSPA7* and *HSPA6* are highly homologous genes. The *HSPA7* was long proposed to not be translated in a functional protein [29] but recently it was suggested to encode for the HSP70B protein [30]. On the other hand, *HSPA6*, which encodes for HSP70B’, is hardly studied because it is not found in mice and rats. However, as previously reported in an in vitro study performed on the *SH-SY5Y* cells, HSP70B’ was found located in the nuclear speckles along with several transcription factors. In compliance with our analysis, the authors associated the protein expression with the recovery from stress [31]. In addition, Shorbagi et al. showed that *HSPA6* has a more dynamic and prolonged association with centrioles in neurons than *HSPA1A* [32]. Interestingly, we found *HSPH1* and *HSPA4L*, members of the Hsp70 superfamily that are properly of the Hsp110s subfamily, to be upregulated. The Hsp110s family proteins are co-chaperones and do not act directly in stress. They assist the activity of the HSP70s, promoting nucleotide exchange [33]. For this reason, we suppose that the genes that encode for Hsp110s in our analysis play an important role in MS. The *HSPH1* gene, also named *HSP105*, was found to support the previously mentioned HSP70B’ (*HSPA6* gene) in other neurodegenerative diseases [34]. *HSPH1* seems also able to act as negative regulator of the activity of Hsp70, but only if it is expressed in complex with Hsc70, encoded by *HSPA8* gene, that is not expressed in our transcriptomes [35]. In addition, Kern et al. showed that *HSPH1* interacts with Nogo-A protein, a myelin-associated inhibitor highly expressed in the myelin sheath [36]. Very few is known about *HSPA2* in somatic cells, but it seems to be involved in cell growth [37]. It is the only HSP70 downregulated in our results.

HSPs have been reported to exert a protective role in different neurodegenerative disorders that are characterized by the accumulation of misfolded proteins and their aggregates, such as Alzheimer’s disease, Parkinson’s disease, amyotrophic lateral sclerosis, Huntington’s disease. Indeed, HSPs represent the main molecular chaperones in cells, that act to mediate the proper folding of proteins, during conditions of stress. For this reason, it was reported that different stress conditions induce their expression [38].

In line with our study, when the HSP70s are drastically overexpressed they can be released from the cell into the milieu and bind to the T cell. The process is interpreted as a warning signal that instigates the reaction of the immune system. Extracellular HSPs also play a role in immunosurveillance through the transport of intracellular antigens to immune cells. The extracellular function of HSPs in the CNS may be summarized as: immune response mediator, antigenic adjuvant, and trigger of antigen-presenting cell maturation and innate immunity. Interacting with cell surface receptors, extracellular HSPs caused the increase in proinflammatory cytokines and chemokines and the activation of dendritic cells, inducing immune responses. Moreover, extracellular HSPs have a role in acquired immunity where they can induce the antigen presentation of the binding peptides [6,7]. The main HSP involved in the immune response is *HSPA1A*, that is expressed inside and around MS lesions, and seems to have a role in the triggering or propagation of the immune response due to its capacity to act as a proinflammatory cytokine [7]. The genes *HSPA1A*, *HSPA1B* and *HSPA6*, members of the HSP70, take part in the process of antigen presentation along with *HSP90AB1*, a member of the Heat Shock Protein 90. Interestingly, the overexpression of the HSP70 seems to promote the myelin autoantigen presentation. Mycko et al. suggest that Major Histocompatibility Complex (MHC) class II is the way used by the organism to increase this antigen presentation [39]. In compliance with this study, our results show that *HLA-DQB1* and *HLA-DRB5* genes, a component of the MHC class II, are overexpressed in the optical chiasm. Moreover, *HLA-DQB1* is upregulated also in corpus callosum, hippocampus and internal capsule. Luckey et al. hypothesized that the interaction between HLA-DR and HLA-DQ predisposes to the MS in a transgenic mice study [40]. The involvement of HLA in MS is supported by different studies that indicated an increased MS susceptibility is associated primarily with some HLA-DRB1 alleles, but also other alleles like HLA-DRB5 may influence the risk. In particular, HLA-DRB1*1501 is a DRB1 allele is considered the primary genetic risk factor for MS [41]. Elevated expression of HLA-DRB5 was found in MS and its expression was associated to the HLA-DRB1*1501 allele [42]. However, our results do not show any statistically significant difference for HLA-DRB1. Moreover, in order to preprocess the antigens, GILT and Cathepsin B, encoded by *IFI30* and *CTSB*, are required in the endosomes. Both the genes are upregulated in our results and in particular the activity of cathepsin B was found to be increased in MS patients by Bever et al. [43]. In addition, MHCII promotes the pathogenesis in the autoimmune diseases through the differentiation of Th-cells [44]. Our results show, in the optic chiasm, the overexpression of *NCK1*. This gene encodes for the cytoplasmic protein NCK1, that is involved in the amplification of T cell signal through the interaction with T cell receptor [45]. Moreover, we found the overexpression of *STAT6* gene, involved in JAK/STAT cascade, that is in line with the study of Hatami et al., where it seems to promote the Th2 cell development [46].

Our results show also *IL1R1* and *IL21R*, encoding for the receptor of interleukin-1 and -21 respectively, that are upregulated in the internal capsule and hippocampus. This is confirmed by Sha et al. and Ghalamfarsa et al., who have already shown that both the interleukins are implicated in the immunopathogenesis of MS through the development of the Th17 cells [47,48]. In addition, Sha et al. demonstrated that the differentiation of the Th17 cells through interleukin receptor 1 is mediated by the interleukin regulatory factor 4, encoded by *IRF4* [49]. We also confirm a role of B cells in MS. Indeed, in line with the study, our results show the overexpression of *IRF4* in corpus callosum and optic chiasm. Xu suggests that *IRF4* is also necessary for the development of B cells [50]. Interestingly, the *CD79A* is very upregulated in corpus callosum and in optic chiasm. It is a cluster of differentiation of B cells and a target in the therapy of several autoimmunity diseases as well as MS [51]. *VAV3*, *BCL10* and *IFNGR2* are also upregulated in optic chiasm. *VAV3* encodes for a guanine nucleotide exchange factor protein that plays a role in the regulation of B cell responses, regulating the downstream events [52]. *BCL10* encodes for the B-cell lymphoma/leukemia 10 protein that, in compliance with our data, is a good candidate in the MS susceptibility due to its relevance in the immune activation. Indeed, this protein takes part in the CBM signalosomes complex that amplifies and mediates both positive and negative regulatory signals [53,54]. Very little is known about leucocyte immunoglobulin-like receptors but in the internal capsule, one of them is quite overexpressed, *LILRA6,* and we think it could have an important role in MS. In general, the leucocyte immunoglobulin-like receptor A seems to activate the immune response and for this reason it could be involved in autoimmune diseases [55]. In addition, *IFNGR2* encodes for interferon γ receptor 2 signaling chain. Regis et al. suggest that this receptor is usually overexpressed in an inflammatory environment [56]. 

Our results show also the downregulation of *MAPK9*, encoding for JNK2 protein, the absence of which was associated with the hyperproliferation of the CD8+ T cells [57]. Moreover, the result is in line with the upregulation of *TAPBP*, encoding for tapasin protein, required to increase the affinity with MHC class I for the activation of the immunogenicity [58]. It is noteworthy that our results show that the genes belonging to the pathways that we took into consideration for the immune system are not statistically differentially expressed in frontal and in parietal cortex of MS patients compared to healthy controls. An explanation for this may be the presence in major number of the nuclei in the cortex, while the axons that are surrounded by the myelin are present in a lower percentage. Moreover, different mechanisms are involved in cortical pathology, including meningeal inflammation, B-cell follicle-like structures and oxidative stress [59]. 

A final consideration regards the number of genes in HSP70s against the immune system and their level of expression, as reported in Figure 2. Indeed, in corpus callosum (Figure 2A), hippocampus (Figure 2B) and internal capsule (Figure 2C) the expression of the HSP70s is higher than the expression of the immune system, while in the optic chiasm (Figure 2D) the immunity has a bigger relevance. In the cortex, there is no activation of the immune system (Figure 2E,F).

## 5. Conclusions

The immune system is undoubtedly involved in the pathogenesis of the MS but the trigger causes are not completely clear. In this study, we showed that *HSPA1A*, *HSPA1B*, *HSPA7*, *HSPA6*, *HSPH1* and *HSPA4L,* encoding for HSP70s, are significantly upregulated in corpus callosum, hippocampus, internal capsule, optic chiasm, and frontal or parietal cortex, between healthy individuals and MS patients. Commonly, HSPs are considered as protective if overexpressed in neurodegenerative disorders. On the contrary, in autoimmune diseases, including MS, HSP70s may have a detrimental role, causing the exacerbation and the promotion of the immune system response against the myelin autoantigen. 

However, the limits of this study are the restricted patient number and the lack of RRMS cases among them. For this reason, further studies are needed to confirm the results in a larger cohort and to evaluate if the results depend on the disease course.

## Figures and Tables

**Table 1:** Genes belonging to the Heat Shock Proteins 70.

Gene	Name	Healthy Expression	Patient Expression	Fold Change	*q*-Value
Corpus callosum					
HSPA7	heat shock protein family A (Hsp70) member 7 (pseudogene)	71.61	335.48	2.23	1.03 × 10^−2^
HSPA6	heat shock protein family A (Hsp70) member 6	135.30	3854.91	4.83	3.51 × 10^−5^
HSPA4L	heat shock protein family A (Hsp70) member 4 like	927.82	3203.44	1.79	3.24 × 10^−3^
HSPA1B	heat shock protein family A (Hsp70) member 1B	8385.32	36140.05	2.11	2.75 × 10^−2^
HSPA1A	heat shock protein family A (Hsp70) member 1A	9503.44	40458.48	2.09	3.73 × 10^−2^
HSPH1	heat shock protein family H (Hsp110) member 1	4420.35	19934.90	2.17	3.57 × 10^−3^
Hippocampus					
HSPA1B	heat shock protein family A (Hsp70) member 1B	2504.61	20344.57	3.02	3.19 × 10^−3^
HSPA1A	heat shock protein family A (Hsp70) member 1A	2741.64	21590.48	2.98	7.50 × 10^−3^
Optic chiasm					
HSPA2	heat shock protein family A (Hsp70) member 2	17822.56	4332.32	−2.04	1.27 × 10^−10^
HSPA7	heat shock protein family A (Hsp70) member 7 (pseudogene)	174.04	687.19	1.98	3.87 × 10^−2^
HSPH1	heat shock protein family H (Hsp110) member 1	5894.37	22661.93	1.94	4.65 × 10^−3^
HSPA1B	heat shock protein family A (Hsp70) member 1B	11058.84	40687.72	1.88	1.61 × 10^−2^
HSPA1A	heat shock protein family A (Hsp70) member 1A	10798.31	33538.69	1.64	3.82 × 10^−2^
HSPA6	heat shock protein family A (Hsp70) member 6	524.15	7134.02	3.77	8.86 × 10^−4^
Internal capsule					
HSPA1B	heat shock protein family A (Hsp70) member 1B	1847.82	22464.49	3.60	1.60 × 10^−4^
HSPA1A	heat shock protein family A (Hsp70) member 1A	2756.75	27201.29	3.30	5.84 × 10^−5^
HSPA7	heat shock protein family A (Hsp70) member 7 (pseudogene)	27.79	286.33	3.33	3.05 × 10^−3^
Frontal Cortex					
HSPA1B	heat shock protein family A (Hsp70) member 1B	1490.03	13373.55	3.17	6.63 × 10^−3^
HSPA1A	heat shock protein family A (Hsp70) member 1A	1670.50	14461.95	3.11	1.24 × 10^−2^
HSPA7	heat shock protein family A (Hsp70) member 7 (pseudogene)	17.54	178.84	3.37	3.31 × 10^−2^
Parietal Cortex					
HSPA1B	heat shock protein family A (Hsp70) member 1B	1727.28	18950.37	3.46	3.64 × 10^−3^
HSPA1A	heat shock protein family A (Hsp70) member 1A	2061.80	23408.21	3.51	5.67 × 10^−3^
HSPA7	heat shock protein family A (Hsp70) member 7 (pseudogene)	12.57	190.93	3.93	3.64 × 10^−3^
HSPA6	heat shock protein family A (Hsp70) member 6	36.82	1185.20	5.02	6.19 × 10^−3^

The HUGO Gene Nomenclature Committee website provided the genes in column Gene and the associated protein name in column Name. Health Expression and Patient Expression columns represent, for healthy individuals and MS patients, respectively, the level of gene expression. The column Fold Change highlights the difference between the expression level of the gene computed by log2 transformation and the q-Value column shows that the genes differences are statistically significant (*q* < 0.05).

**Table 2:** Genes involved in the Immune System.

Gene	Name	Healthy Expression	Patient Expression	Fold Change	*q*-Value
Corpus callosum					
TAPBP	TAP binding protein	2254.66	3431.10	0.61	1.54 × 10^−2^
IRF4	interferon regulatory factor 4	17.70	64.86	1.88	3.12 × 10^−3^
CTSB	cathepsin B	8640.73	17124.86	0.99	3.08 × 10^−2^
IFI30	IFI30 lysosomal thiol reductase	13.63	37.46	1.48	4.26 × 10^−2^
HSP90AB1	heat shock protein 90 α family class B member 1	23769.44	45751.07	0.94	4.21 × 10^−2^
HLA-DQB1	major histocompatibility complex, class II, DQ β 1	249.73	1016.29	2.03	1.39 × 10^−2^
CD79A	CD79a molecule	6.88	83.66	3.59	3.33 × 10^−6^
Hippocampus					
IL21R	interleukin 21 receptor	10.40	112.12	3.42	1.92 × 10^−2^
HLA-DQB1	major histocompatibility complex, class II, DQ β 1	143.49	643.50	2.17	4.96 × 10^−2^
Optic chiasm					
IRF4	interferon regulatory factor 4	35.94	138.01	1.95	2.12 × 10^−3^
VAV3	vav guanine nucleotide exchange factor 3	90.27	174.52	0.96	2.62 × 10^−2^
CTSB	cathepsin B	13960.93	26669.15	0.93	4.79 × 10^−3^
NCK1	NCK adaptor protein 1	488.05	708.92	0.54	2.37 × 10^−2^
STAT6	signal transducer and activator of transcription 6	2661.12	4805.78	0.85	1.76 × 10^−2^
HLA-DRB5	major histocompatibility complex, class II, DR β 5	1051.38	3271.14	1.64	1.34 × 10^−2^
HSP90AB1	heat shock protein 90 α family class B member 1	26497.02	45815.97	0.79	1.77 × 10^−2^
HLA-DQB1	major histocompatibility complex, class II, DQ β 1	577.34	1919.98	1.73	2.19 × 10^−2^
IFNGR2	interferon γ receptor 2	1096.46	1814.94	0.73	7.04 × 10^−3^
TAPBP	TAP binding protein	3067.78	4365.48	0.51	3.09 × 10^−2^
BCL10	BCL10 immune signaling adaptor	233.04	378.67	0.71	4.26 × 10^−2^
CD79A	CD79a molecule	9.29	210.76	4.48	1.31 × 10−^11^
Internal capsule					
HLA-DQB1	major histocompatibility complex, class II, DQ β 1	100.94	610.62	2.60	3.47 × 10^−2^
LILRA6	leukocyte immunoglobulin like receptor A6	6.43	40.55	2.60	2.79 × 10^−2^
MAPK9	mitogen-activated protein kinase 9	2429.45	1365.84	−0.83	4.39 × 10^−2^
IL1R1	interleukin 1 receptor type 1	345.25	1046.19	1.60	4.00 × 10^−2^
Frontal Cortex					
No gene.					
Parietal Cortex					
No gene.					

The HUGO Gene Nomenclature Committee website provided the genes in column Gene and the associated protein name in column Name. Health Expression and Patient Expression columns represent, for healthy individuals and MS patients, respectively, the level of gene expression. The column Fold Change highlights the difference between the expression level of the gene computed by log2 transformation and the *q*-Value column shows that the genes differences are statistically significant (*q* < 0.05).

## Supplementary Materials

The following are available online at https://www.mdpi.com/2073-4425/11/6/615/s1, Table S1: Code used in the analysis, Table S2: Differentially Expressed Genes in Corpus Callosum, Table S3: Differentially Expressed Genes in Hippocampus, Table S4: Differentially Expressed Genes in Optic Chiasm, Table S5: Differentially Expressed Genes in Internal Capsule, Table S6: Differentially Expressed Genes in Frontal Cortx, Table S7: Differentially Expressed Genes in Parietal Cortex.
