# Supplementary material for: Could the Heat Shock Proteins 70 Family Members Exacerbate the Immune Response in Multiple Sclerosis? An in Silico Study
Source: Genes (Basel). 2020 Jun 3;11(6):615. doi: 10.3390/genes11060615 (PMC7348765; doi:10.3390/genes11060615)
Supplement: Supplementary file 1 [file genes-11-00615-s001.zip › Table S1.docx]

**Table S1.** Code used in the analysis.

**>>> BASH**

#For each sample:

java -jar trimmomatic SE -phred33 $input_R1 $output_dir/$R1_name ILLUMINACLIP:adapters.fasta:2:30:10 LEADING:30 TRAILING:28 SLIDINGWINDOW:4:28 MINLEN:35
STAR --genomeDir $resources --readFilesIn $output_dir/$R1_name --outFileNamePrefix $output_dir/ --outFilterIntronMotifs RemoveNoncanonical --outSAMtype BAM SortedByCoordinate

htseq-count -s reverse -i gene_name -f bam $output_dir /Aligned.sortedByCoord.out.bam resources/Homo_sapiens.GRCh37.75.gtf > Aligned.sortedByCoord.count.txt

**>>> R code (version 3.6.0)**

library("DESeq2")

groups <- c("internalcapsule", "hippocampus", "opticchiasm", "corpuscallosum", "frontalcortex", "parietalcortex")

healthy.subpath <- "healthy/output"

sick.subpath <- "sick/output"

groups.list <- list()

for(group in groups){

main.path <- paste(output.path, group, sep="")

healthy.sample <- list.files(paste(main.path, "/", healthy.subpath, sep=""))

sick.sample <- list.files(paste(main.path, "/", sick.subpath, sep=""))

healthy.table <- data.frame(sampleName = healthy.sample, fileName = paste(healthy.subpath, "/", healthy.sample, "/Aligned.sortedByCoord.count.txt", sep=""), condition = "CTR")

sick.table <- data.frame(sampleName = sick.sample, fileName = paste(sick.subpath, "/", sick.sample, "/Aligned.sortedByCoord.count.txt", sep=""), condition = "MS")

samples.table <- rbind(healthy.table, sick.table)

ddsHTSeq <- DESeqDataSetFromHTSeqCount(sampleTable = samples.table, directory = main.path, design= ~ condition)

ddsHTSeq$condition <- relevel(ddsHTSeq$condition, ref = "CTR")

dds <- DESeq(ddsHTSeq)

keep <- rowSums(counts(dds)) >= 10

dds <- dds[keep,]

norm.counts <- counts(dds, normalized=TRUE)

norm.counts.CTR<-rowMeans(norm.counts[,1:length(healthy.sample)])

norm.counts.MS<-rowMeans(norm.counts[,length(healthy.sample)+1:length(sick.sample)])

res.corrected <- results(dds, pAdjustMethod = "BH", alpha=0.05)

res.final <- subset(data.frame(norm.counts.CTR, norm.counts.MS, res.corrected), padj<0.05)

res.tableable <- data.frame(round(subset(res.final, select=c("norm.counts.CTR", "norm.counts.MS", "log2FoldChange")), digits=2), formatC(res.final$padj,format="e",digits=2))

colnames(res.tableable)<-c("Control", "Patient", "Fold Change", "q-Value")

previous.names <- names(groups.list)

groups.list <- append(groups.list, list(res.tableable))

names(groups.list) <- c(previous.names, group)

}
